# Facile Construction of Porous ZnMn_2_O_4_ Hollow Micro-Rods as Advanced Anode Material for Lithium Ion Batteries

**DOI:** 10.3390/nano13030512

**Published:** 2023-01-27

**Authors:** Yuyan Wang, Senyang Xu, Yamin Zhang, Linrui Hou, Changzhou Yuan

**Affiliations:** School of Materials Science & Engineering, University of Jinan, Jinan 250022, China

**Keywords:** porous ZnMn_2_O_4_, hollow micro-rods, co-precipitation synthesis, anode materials, Li-ion batteries

## Abstract

Spinel ZnMn_2_O_4_ is considered a promising anode material for high-capacity Li-ion batteries due to their higher theoretical capacity than commercial graphite anode. However, the insufficient cycling and rate properties seriously limit its practical application. In this work, porous ZnMn_2_O_4_ hollow micro-rods (ZMO HMRs) are synthesized by a facile co-precipitation method coupled with annealing treatment. On the basis of electrochemical analyses, the as-obtained samples are first characterized by X-ray diffraction, X-ray photoelectron spectroscopy, transmission electron microscopy, and scanning electron microscopy techniques. The influences of different polyethylene glycol 400 (PEG 400) additions on the formation of the hollow rod structure are also discussed. The abundant multi-level pore structure and hollow feature of ZMO HMRs effectively alleviate the volume expansion issue, rendering abundant electroactive sites and thereby guaranteeing convenient Li^+^ diffusion. Thanks to these striking merits, the ZMO HMRs anode exhibits excellent electrochemical lithium storage performance with a reversible specific capacity of 761 mAh g^−1^ at a current density of 0.1 A g^−1^, and a long-cycle specific capacity of 529 mAh g^−1^ after 1000 cycles at 2.0 A g^−1^ and keep a remarkable rate capability. In addition, the assembled ZMO HMRs-based full cells deliver an excellent rate capacity, and when the current density returns to 0.05 A g^−1^, the specific capacity can still reach 105 mAh g^−1^ and remains at 101 mAh g^−1^ after 70 cycles, maintaining a material-level energy density of approximately 273 Wh kg^−1^. More significantly, such striking electrochemical performance highlights that porous ZMO HMRs could be a promising anode candidate material for LIBs.

## 1. Introduction

With the ever-increasing requirements for Li-ion batteries (LIBs), it is crucial to develop an anode material with a high specific capacity [1,2,3,4,5]. Tarascon’s group applied transition metal oxide to the anode materials for LIBs for the first time in 2000. Transition metal oxides (TMOs), as a new type of anode material, are considered an attractive substitute for commercialized graphite anode material due to their high theoretical specific capacity [6]. Among them, Mn-based transition metal oxides have quickly attracted researchers’ attention due to their environmental friendliness, low cost, and abundant element reserves [7,8,9,10,11]. However, the large volume expansion during the lithiation/delithiation process leads to the degradation of the electrochemical performance, thus hindering its practical application.

Recent studies have found that compounding the TMO with other metals can effectively improve the volume expansion problem through the synergistic effect [12,13,14]. Among these binary metal oxides, ZnMn_2_O_4_ (ZMO) attracted extensive attention in view of its low cost, abundant element reserves, low operating voltage platform, environmental friendliness, and simple preparation method [15,16]. In addition, Zn and Mn elements in ZMO have different potentials (1.2 V and 1.5 V vs. Li^+^/Li), which can synergistically interact during the lithiation/delithiation process, thereby reducing the stress concentration in the Li^+^ intercalation and deintercalation process and improving the electrode pulverization phenomenon [2,17]. Moreover, the lithium storage process of ZMO involves both the conversion reaction and alloying reactions (Zn in ZMO can alloy with Li to form LiZn, offering extra specific capacity) [5,18,19]. Therefore, the theoretical specific capacity of ZMO is higher than that of other metal oxides.

As established before, porous structures or novel nanostructures can effectively relieve the volume expansion and grant the material superior electrochemical performance [17,20,21,22,23,24]. Rong et al. [23] synthesized hierarchical porous ZMO microspheres (MSs) constructed with sub-nanoparticles, and tested as anode materials for LIBs, the initial specific discharge and charge capacity were 1238 and 779 mAh g^−1^, respectively. Zhang et al. [17] successfully prepared the hollow core-shell ZMO MSs by a solvothermal carbon template method and then an annealing process. Electrochemical properties of ZMO MSs as anode materials for LIBs show that ZMO MSs can provide a reversible specific capacity of 856 mAh g^−1^ at 0.2 A g^−1^. Lou’s group [24] synthesized ZMO hollow MSs with a sphere-in-sphere structure by a facile two-step method. These ZMO hollow MSs showed excellent cycling stability and good rate performance when used as anode materials for LIBs. The excellent electrochemical performance mentioned above can be attributed to the hierarchical porous/hollow core-shell structure, which facilitates the penetration of the electrolyte and thus shortens the transport distance of Li^+^ and effectively accommodates the volume expansion during lithiation/delithiation cycles, enabling better utilization of active materials and easily facilitated diffusion of electrolytes into and out of electrode materials.

Herein, the porous ZMO hollow micro-rods (HMRs) with abundant pore structures are synthesized by a facile co-precipitation method and annealing treatment. The content of PEG 400 in the mixed solution plays a key role in the formation of the hollow structure. As expected, benefiting from the abundant multi-level pore structure and hollow structure, the ZMO HMRs anode exhibits excellent electrochemical lithium storage performance with a reversible specific capacity, a remarkable rate capability, as well as a long-cycle specific capacity. In addition, the assembled ZMO HMRs-based full cells deliver an excellent rate capacity and maintain a material-level energy density of approximately 273 Wh kg^−1^.

## 2. Materials and Methods

### 2.1. Material Preparation

All the chemicals were directly used without further treatment. Firstly, 1.5 mmol of oxalic acid dihydrate (H_2_C_2_O_4_·2H_2_O) was dissolved into 20 mL of absolute ethanol under magnetic stirring. After complete dissolution, 30 mL of polyethylene glycol 400 (PEG 400) was added to the above solution, and after being stirred evenly, 1 mmol of zinc chloride (ZnCl_2_) and 1 mmol of manganese chloride tetrahydrate (MnCl_2_·4H_2_O) were added and stirred for 10 min, then 10 mL of deionized water was added stirred for 4 h at room temperature (RT) until the solution turned a milky white color. After that, the final solution was filtered out, washed with deionized water and absolute ethanol every three times, and then dried in an oven for 12 h to obtain the white powder products. The collected white powder was calcinated in the furnace at 400 °C for 2 h with a heating rate of 1 °C min^−1^ to obtain the final brown powder, which was named ZMO HMRs.

In addition, in order to explore the effect of metal salts on the structure of the products, the original chloride salt (ZnCl_2_ + MnCl_2_·4H_2_O) was replaced with zinc acetate dihydrate (Zn(CH_3_COO)_2_·2H_2_O), manganese acetate tetrahydrate (Mn(CH_3_COO)_2_·4H_2_O), zinc nitrate hexahydrate (Zn(NO_3_)_2_·6H_2_O), and manganese nitrate (Mn(NO_3_)_2_, a 50 wt.% solution) with the same molar ratio of Zn^2+^ and Mn^2+^ of 1:2, and the other conditions were kept the same as the above steps. The obtained products were named ZMO-AC and ZMMO-NO, respectively. To investigate the influence of different addition contents of PEG 400 on the hollow rod structure, the products were prepared with different PEG 400 contents of 0, 10, 20, and 40 mL, named ZMO-0, ZMO-10, ZMO-20, and ZMO-40, respectively, while other conditions were the same as ZMO HMRs.

### 2.2. Characterization

The crystal phase structure of the obtained samples was characterized by Rigaku UItimaIV X-ray powder diffraction (XRD, Cu-Kα radiation from 2*θ* = 10 to 80°). The chemical composition on the surface of the sample was analyzed by X-ray photoelectron spectroscopy (XPS, Thermo ESCALAB 250Xi), and the X-ray source was an Al Kα monochromatic beam (1486.6 eV). The micromorphology and structure were observed by field-emission electron microscopy (FESEM, SIGAM 500), transmission electron microscopy (TEM), high-resolution TEM (HRTEM), and selected area electron diffraction (SAED) (JEOL JEM-2100). The thermogravimetric analysis (TGA) and differential scanning calorimetry (DSC) data were measured by a thermogravimetric analyzer (DTG 60H, Shimadzu Company, Kyoto, Japan) in an oxygen atmosphere at a heating rate of 10 °C min^−1^, and the specific surface area was measured using the Brunauer–Emmett–Teller (BET) method (Autosorb iQ, Canta Instrument Company, Boynton Beach, FL, USA). 

### 2.3. Electrochemical Measurements

The electrochemical properties of as-synthesized samples were investigated by detecting the CR2032-type coin cell. Firstly, the working electrodes were fabricated by mixing 70 wt.% ZMO HMRs, 20 wt.% acetylene black, and 10 wt.% sodium hydroxymethylcellulose (CMC) in a weight mortar in deionized water to form a uniform slurry with moderate viscosity. Then, the slurry was coated onto the copper foil and dried in a vacuum at 110 °C for 12 h to completely remove the moisture. After being cooled to RT, the copper foil was cut into a circular piece with a diameter of 12 mm and the loading of the active materials was approximately 1.2 mg. The cells were assembled in a glove box (a high-purity argon environment, water/oxygen content < 0.1 ppm) with Li foil as the counter/reference electrode, a Celgard 2400 polymer film as the separator, LiPF_6_ as the electrolyte (1 M, EC:DMC:EMC = 1:1:1 in volume), and a charge and discharge test in the CT2001A Land system (Land Instrument Inc., Wuhan, China). Electrochemical impedance spectroscopy (EIS) was measured from a frequency range of 0.01 to 100 kHz. Cyclic voltammetry (CV) was conducted between 0.01 and 3.0 V at a sweep rate of 0.1 mV s^−1^. Both EIS and CV tests were carried out on the Ivium electrochemical workstation (IviumStat.h, Ivium Technologies BV, Eindhoven, The Netherlands) at RT.

For full-cell preparation, the cathode electrode was fully mixed with 80 wt.% commercial LiNi_0.8_Co_0.1_Mn_0.1_O_2_ (LNCM), 10 wt.% acetylene black, and 10 wt.% polyvinylidene fluoride (PVDF) with an appropriate amount of 1-methyl-2-pyrrolidone (NMP) to form a uniform slurry, which was pasted evenly on Al foil and dried in a vacuum oven at 110 °C for 12 h, and the electrode was cut into a circular piece with 12 mm after being cooled to RT. Before preparing the full battery, the anode electrode was pre-lithium-treated to eliminate the irreversible capacity loss of the first cycle. The capacity ratio of the cathode to anode electrodes was 1: 1.1–1.5.

## 3. Results

### 3.1. Morphology and Structures

**Scheme 1:**
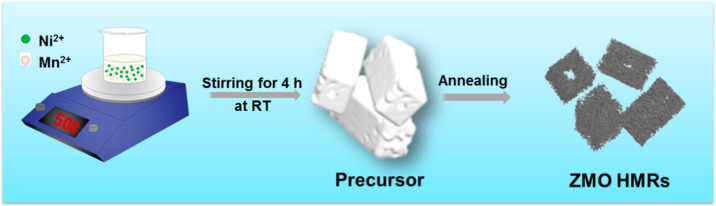
Schematic illustration for the formation of porous ZMO HMRs.

**Figure 1:**
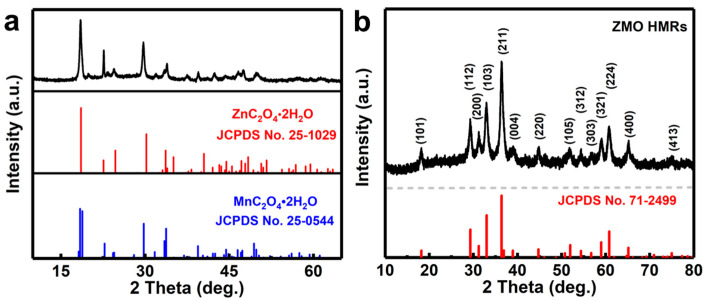
XRD patterns of the (**a**) ZnC_2_O_4_-MnC_2_O_4_·2H_2_O precursor and (**b**) ZMO HMRs.

The porous ZMO HMRs were prepared by a simple co-precipitation process and subsequent heat treatment, as schematically illustrated in Scheme 1. As an important intermediate for porous ZMO HMRs, the precursor obtained after the co-precipitation process was first tested by XRD. All the diffraction peaks can be perfectly indexed as a mixture of ZnC_2_O_4_·2H_2_O (JCPDS No. 25-1029) and MnC_2_O_4_·2H_2_O (JCPDS No. 25-0544) (Figure 1a). After the calcination process at 400 °C for 2 h, the obtained products can be well indexed as ZMO (JCPDS No. 71-2499) with a tetragonal spinel structure of a space group of *I*4_1_/*amd* (Figure 1b) [16] and the Rietveld refinement of the XRD data in Figure S1, while Table S1 shows that both the R_wp_ and R_p_ are less than 10% (R_wp_ = 3.74% and R_p_ = 2.56%). The unit cell parameters for ZMO HMRs were calculated to be *a* = *b* = 5.652(3) Å, *c* = 8.998(9) Å, and *V* = 287.612 Å^3^, respectively, indicating the formation of the high phase-pure ZnMn_2_O_4_. The corresponding morphology and structure are characterized by FESEM and TEM in Figure S2. Visually, the precursor is a hollow rod-like structure with an uneven rod surface, and the average diameters of micron rods were approximately 0.2–1.2 µm and approximately 1.5–2.5 µm in overall length (Figure S2a). At high magnification, it can be clearly seen that the precursor has a pore structure at the top face of the rod (Figure S2b). To further verify the hollow structure of the precursor, TEM tests were performed as shown in Figure S2c–d. Obviously, the precursor exhibits an obvious hollow structure (Figure S2c), which is consistent with the FESEM observations. The enlarged view (Figure S2d) indicates that the precursor consisted of a large number of vesicle structures with different sizes on the rod’s outer wall.

To explore the thermal behavior of the porous ZMO HMRs during the annealing process, the precursor ZnC_2_O_4_-MnC_2_O_4_·2H_2_O was analyzed by TGA-DSC tests at 30–600 °C. As shown in Figure S3, the first weight loss (the corresponding DSC curve shows an endothermic peak) between 120 and 170 °C can be ascribed to the volatilization of crystal water in the precursor, and the second largest mass loss of approximately 35.1% occurs at 280–330 °C, which is caused by the decomposition of oxalic acid and the formation of ZMO [25]. There was no obvious change after 400 °C, which indicates that the sample is almost completely decomposed and no other reaction occurs.

**Figure 2:**
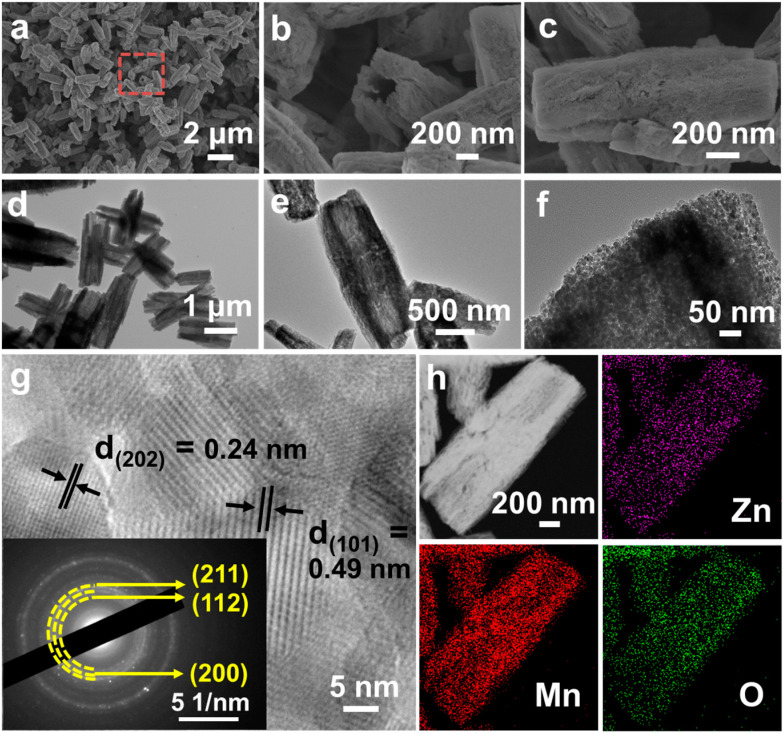
(**a**–**c**) FESEM images, (**d**,**e**) TEM images, (**f**,**g**) HRTEM images, SAED pattern (the inset in panel G), and (**h**) scanning TEM and corresponding EDS elemental (Zn, Mn, and O) mapping images of ZMO HMRs.

The morphology and microstructure of the as-obtained ZMO HMRs were further studied via SEM and TEM. It can be clearly seen that the morphology still maintained a good and complete rod-like structure after annealing, as shown in Figure 2a, and a magnified SEM image (Figure 2b) taken from the orange region in Figure 2a shows the pore structure with a rough surface. TEM images in Figure 2d,e clearly exhibit the inside hollow structure of the rod, and the ZMO HMRs still maintained a hollow structure even after 400 °C heat treatment. ZMO HMRs are formed by the accumulation of nanocrystalline, and there are abundant pore structures between the grains. The porous hollow structure can effectively alleviate the volume change during the lithiation/delithiation process and significantly improve the cycle stability of the batteries [17,23,26]. As can be seen, the well-resolved lattice fringes in Figure 2g from the HRTEM image indicates interplanar distances of approximately 0.24 nm and 0.49 nm, which match the (202) and (101) plane spacing of the tetragonal ZMO HMRs. The diffraction rings also revealed the polycrystalline nature and can be indexed to the (211), (112), and (200) planes of ZMO, which is consistent with the XRD test results. In addition, randomly selected ZMO HMRs are scanned by energy-dispersive X-ray (EDS) elemental mapping. As shown in Figure 2h, the Zn, Mn, and O elements are evenly distributed in the hollow rod structure. In addition, to further confirm the pore structure of ZMO HMRs, Brunauer–Emmett–Teller (BET) N_2_-sorption measurements are displayed in Figure S4. The ZMO HMRs exhibit a high BET-specific surface area of 89.6 m^2^ g^−1^, an average pore size of 15.8 nm, and a pore volume of 0.35 cm^3^ g^−1^. The abundant pore structure can significantly improve the electroactive sites and ion transport rate on the surface of the electrode material. The porous hollow structure effectively alleviates the volume change during the Li^+^ insertion/extraction process and significantly improves the cycle stability of the battery.

**Figure 3:**
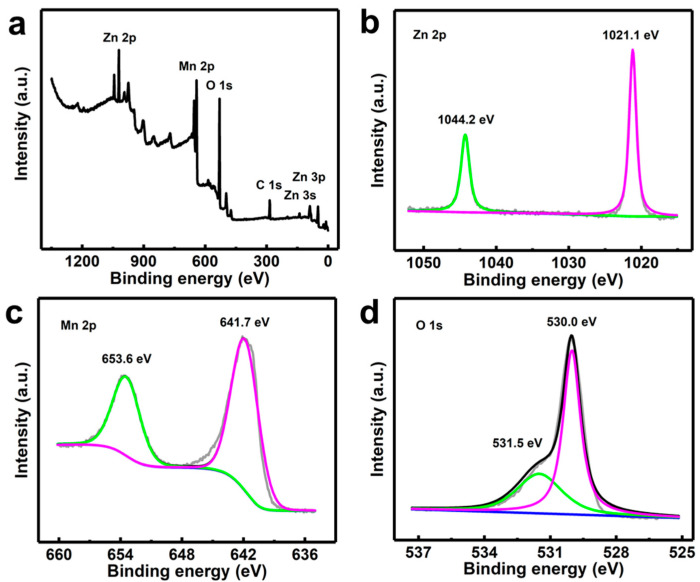
(**a**) XPS survey and high-resolution (**b**) Zn 2p, (**c**) Mn 2p, and (**d**) O 1s spectra of the ZMO HMRs.

To further confirm the detailed element composition and valence state of the ZMO HMRs, X-ray photoemission spectroscopy (XPS) studies were carried out as shown in Figure 3. The presence of Zn, Mn, and O in the ZMO HMRs can be observed in the survey XPS spectrum (Figure 3a) and the valence states of the elements are shown in high-resolution XPS spectra in Figure 3b–d. Figure 3b presents the spectrum and corresponding fitting curve of Zn 2P, where the binding energies of 1021.1eV and 1044.2 eV are indexed to Zn 2p_3/2_ and Zn 2p_1/2_, respectively [27]. The difference in spin energy levels between the two peaks (23.1eV) indicates the oxidation state of Zn (II) in the ZMO sample, and the two peaks presented at 641.7 and 653.6 eV in Figure 3c can be ascribed to the Mn 2p_3/2_ and Mn 2p_1/2_ of Mn^3+^ [15,16,23,27]. The spectrum of O 1s (Figure 3d) shows the binding energy positions at 530.0 and 531.5 eV, corresponding to the oxygen in the metal–oxygen bond [28,29] and oxygen in –OH, respectively [30].

### 3.2. Effect of PEG 400 Addition on the Hollow Rod Structure

**Figure 4:**
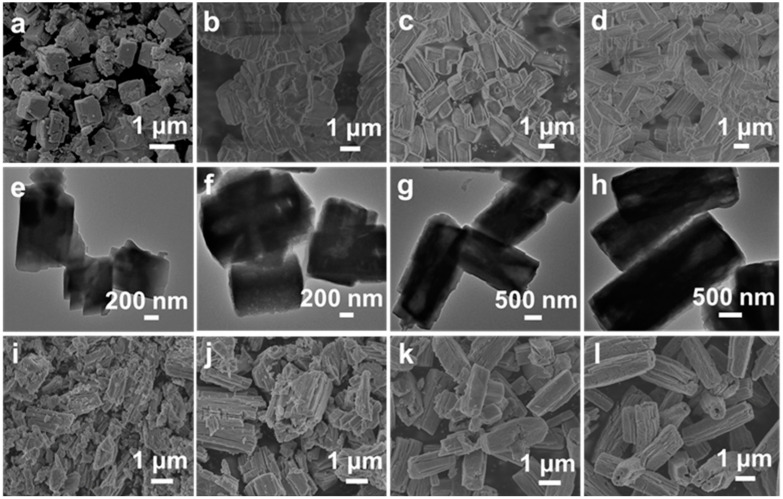
(**a**–**d**) FESEM and (**e**–**h**) TEM images of the ZnC_2_O_4_-MnC_2_O_4_·2H_2_O precursor with different volumes of PEG 400: (**a**,**e**) 0 mL, (**b**,**f**) 10 mL, (**c**,**g**) 20 mL, (**d**,**h**) 40 mL. FESEM images of (**i**) ZMO-0, (**j**) ZMO-10, (**k**) ZMO-20, and (**l**) ZMO-40.

To investigate the different addition contents of PEG 400 on the influence of the hollow rod structure, the morphology of samples obtained by adding different volumes of PEG 400 was tested as shown in Figure 4. Figure 4a–d show the FESEM images of ZnC_2_O_4_-MnC_2_O_4_·2H_2_O precursors prepared with the PEG content of 0 mL, 10 mL, 20 mL, and 40 mL, respectively. When 0 mL of PEG 400 was added to the mixed-solution system (Figure 4a), most of the ZnC_2_O_4_-MnC_2_O_4_·2H_2_O precursors were cuboid structures with different block sizes, and no pore structure can be found at the block ports. When 10 mL of PEG 400 was added to the mixed solution, the pore structures with a small amount at the rod-shaped port can be found (Figure 4b). As the content of PEG 400 increases to 20 mL (Figure 4c), the porous ZMO micro-rods begin to form and grow laterally, and the hollow structure becomes more obvious. When the PEG 400 is further increased to 40 mL (Figure 4d), the rod-like structure can be still maintained, and the pore structure can be observed at the rod-like port. To further explore the internal structure of the rod-like precursor, corresponding TEM tests were carried out and are shown in Figure 4e–h. As shown in Figure 4e, the precursor displays a solid structure without the PEG 400 added. With increasing content of PEG 400, a large number of hollow rod-like structures are observed (Figure 4f,g), but are still not uniform. When the PEG 400 increases to 40 mL (Figure 4h), the hollow rod-like structure is uniform, and the internal cavity structure is still not complete. This result is consistent with the FESEM observation. After annealing the precursors at 400 °C for 2 h, the microstructure of ZMO was significantly damaged when PEG 400 was not present (Figure 4i). The hollow rod-like structure remains intact and gradually lengthened along the axial direction when PEG 400 was added from 10 to 40 mL (Figure 4j–l). PEG is a chain polymer with the -O- and -CH_2_-CH_2_-- bonds, which can adsorb metal ions such as Zn^2+^ and Mn^2+^ [31], and it also can be used as the soft template to easily form a hollow structure.

To explore the effect of metal salts on the chemical composition of the products, the original chloride salt was replaced with acetate salts and nitrate salts, respectively, and the obtained products were analyzed by the XRD tests (Figure S5). The ZMO-AC is perfectly matched with criterion JCPDS No.71-2499 (Figure S5a), which is well indexed to pure ZnMn_2_O_4_ peaks with a JCPDS card. However, the XRD pattern of ZMMO-NO (Figure S5b) shows a clear difference, which is the mixture of ZnMnO_3_ (JCPDS No.19-1461) and MnO_2_ (JCPDS No.42-1316). The corresponding morphology and microstructure of the obtained samples via SEM and TEM images are shown in Figure S6. When acetates were used as metal salt sources, the precursor showed a rod-like structure (an axial length of approximately 1.9µm and a radial width of approximately 200 nm) with a smooth surface (Figure S6a). The ZMO-AC still maintained a complete rod-like structure after annealing treatment (Figure S6b). TEM images in Figure S6c show that the ZMO-AC was stacked by a large number of nanoparticles without an obvious hollow structure. In addition, the morphology of the obtained nitrate precursor sample (Figure S6d) also presents a rod structure with a radial length of approximately 1.8 μm and an axial width of approximately 750 nm. After annealing treatment, the microstructure of the ZMO-NO sample remained intact and did not change significantly (Figure S6e). Similarly, the internal structures of the ZMO-NO samples observed through TEM were still solid structures (Figure S6f). Although the three samples prepared with different salt sources show a rod-like structure, only the chloride salt can obtain a hollow structure. This is because the three metal salts exist in different forms in the solution and grow in different ways with EG as the solvent [32]. Finally, the ZMO-AC is used as the comparison sample.

### 3.3. Electrochemical Characterization

**Figure 5:**
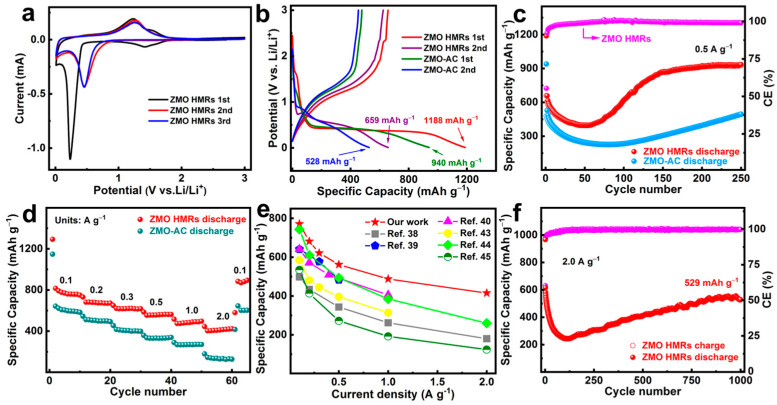
Electrochemical evaluation of the ZMO HMRs and ZMO-AC: (**a**) CVs curves of ZMO HMRs (0.1 mV s^−1^), (**b**) galvanostatic charge–discharge profiles at 0.5 A g^−1^, (**c**) cycling stabilities 0.5 A g^−1^, (**d**) rate capacities, (**e**) comparisons in rate performance between ZMO HMRs and other reported Mn-based metal oxide anodes, and (**f**) cycling performance at 2.0 A g^−1^.

In order to explore the electrochemical performance, various performance tests were carried out in Figure 5. The ZMO HMRs served as the anode materials with Li foil as the counter electrode and 1 M LiPF_6_ as the electrolyte, and ZMO-AC was used as the comparison sample. The cyclic voltammetry (CV) test is presented in Figure 5a at a scanning rate of 0.1 mV s^−1^ with a voltage window of 0.01–3.0 V (vs. Li/Li^+^). During the first cathodic scan process, two obvious reduction peaks can be observed. The first broad peak located at approximately 1.41 V, which vanishes in the following cycles, could be attributed to the reduction of Mn^3+^ to Mn^2+^ and the irreversible reaction of Li^+^ in the electrolyte to form the solid electrolyte interphase (SEI) film [33,34]. The sharp peak centered at 0.24 V can be assigned to the reductive transformation of Zn^2+^ and Mn^2+^ to Zn^0^ and Mn^0^ embedded in the Li_2_O matrix [16,22,26]. During the subsequent anodic process, the weak peak at approximately 0.5 V should be ascribed to the dealloying process of ZnLi and the two oxidation peaks can be attributed to the reversible formation of MnO at approximately 1.23 V and ZnO at approximately 1.53 V, respectively [21,35]. In the following cycles, the reduction peak shift from 1.41 V to 0.45 V can be ascribed to the reduction of ZnO and MnO to metal Zn and Mn, and the oxidation peaks are almost analogous to the first cycle regarding the similar process for anodic cycles [36]. Remarkably, there are no significant changes in the oxidation/reduction process after the first cycle, indicating the high reversibility and desirable cycling stability of the ZMO HMRs electrode. To provide a further in-depth understanding of the intrinsic storage mechanism of our ZMO HMRs electrode, ex situ XRD analysis of the ZMO HMRs over the typical initial charge–discharge profile was conducted at different selected voltages as shown in Figure S7. Apparently, the broad peak at approximately 25° can be ascribed to the C signal from carbon black. During the first lithiation process, the original ZnMn_2_O_4_ can still be maintained with a weak peak at approximately 1.41 V, and the plateau starting from 0.45 V represents the reduction of ZnO (JCPDS NO.: 65-0641) and MnO (JCPDS NO.: 65-0682) to metal Zn and Mn, respectively. However, no obvious XRD peaks of other phases (metallic Mn, Zn, and/or LiZn alloy) can be detected after discharging from 0.31 V to 0.01 V, and this can be ascribed to the nanoparticles’ nature. Similar to the following delithiation process, there are also no obvious diffraction peaks that can be found. This phenomenon can be found in the previous results [16,36,37]. The whole Li^+^ storage process can be summarized in the following equations:

The first lithiation process:

ZnMn_2_O_4_ + 9Li^+^ + 9e^−^ → ZnLi + 2Mn + 4Li_2_O (1)

The following reversible reactions:

Zn + Li_2_O ⇄ ZnO + 2Li^+^ + 2e^−^ (2)

Mn + Li_2_O ⇄ MnO + 2Li^+^ + 2e^−^ (3)

ZnLi ⇄ Zn + Li^+^ + e^−^ (4)

Figure 5b shows the first two galvanostatic charge-discharge curves of the ZMO HMRs and ZMO-AC at a current density of 0.5 A g^−1^. In the first lithiation process, the specific discharge capacity of the ZMO HMRs reaches as high as 1188 mAh g^−1^, which is much higher than the theoretical specific capacity (1008 mAh g^−1^). The extra specific capacity could be ascribed to the formation of the SEI film at the electrode/electrolyte surface and the partial decomposition of the electrolyte in the first lithiation cycle. In the subsequent delithiation process, the initial specific charge capacity reached 658 mAh g^−1^, and the initial Coulombic efficiency (ICE) was 55.3%. During the second lithiation/delithiation cycle, the discharge- and charge-specific capacities of ZMO HMRs electrodes are 659 mAh g^−1^ and 625 mAh g^−1^, respectively, and the CE quickly increases to 94.8% and stabilizes at nearly 100% as shown in Figure 5c. In contrast, the initial discharge/charge specific capacity of the ZMO-AC electrode is only 940/477 mAh g^−1^ along with a smaller ICE of 50.8%, which is much lower than that of ZMO HMRs. The specific capacity is also significantly lower than that of the ZMO HMRs electrode in the second cycle.

Figure 5c comparatively shows the cycling performance of the two different materials at a current density of 0.5 A g^−1^. It can be seen that the specific discharge capacities of the two showed an obvious downward trend in the first 50 cycles, and an upward trend in the following 150 cycles, then slowly increased to 200 cycles and stabilized at 932 mAh g^−1^ after 250 cycles, while the Coulombic efficiency exceeded 100% in the initial dozens of cycles. This trend can be observed for the transition metal oxides, which were primarily due to the reversible formation of the SEI films during the activation of electrode materials [38,39,40]. After that, with the continuous activation of materials, the specific discharge capacity of the electrode material slowly increased, which can be ascribed to the evolution of the original ZnMn_2_O_4_ particles into smaller ZnO and MnO particles during the cycle [21,36,41,42]. Meanwhile, the ZMO HMRs electrode exhibited an excellent reversible capacity with the formation of durable SEI films, and the CE of the sample gradually stabilized at approximately 100% during the following cycling process. As a comparison, ZMO-AC shows a similar trend, but the discharge-specific capacity was only 492 mAh g^−1^ after 250 cycles, further illustrating the excellent electrochemical performance of ZMO HMRs electrodes. To further investigate the stability of the sample, the TEM image of the electrode after 250 cycles is presented in Figure S8. It can be clearly seen that the hollow rod structure is still maintained, which implies the structural stability of the ZMO HMRs during the lithiation/delithiation process, and only approximately 127% expansion can be observed from the thickness of pristine ZMO HMRs electrode, which increases from 110 to 124 µm after 250 cycles at a current density of 0.5 A g^−1^ (Figure S9). The excellent electrochemical lithium storage performance can be attributed to its stable microstructure. The porous hollow structure can effectively relieve the strain caused by the volume change during the cycling process.

In addition to the super reversible capacities, an excellent rate capability was also observed. Figure 5d illustrates the rate performance of the ZMO HMRs and ZMO-AC within a wide current range from 0.1 to 2.0 A g^−1^ in a voltage window of 0.01 to 3.0 V (vs. Li/Li^+^). Remarkably, the ZMO HMRs exhibit the varied reversible specific capacity of 761, 679, 618, 562, 488, 423 mAh g^−1^ with a rate change of 0.1, 0.2, 0.3, 0.5, 1.0, and 2.0 A g^−1^, respectively. When the current density returns to 0.1 A g^−1^, the specific capacity still remains at 884 mAh g^−1^. In contrast, the specific capacity of the ZMO-AC electrode is relatively lower than ZMO HMRs, and the average specific capacity is 605, 504, 406, 333, 268, and 134 mAh g^−1^ under the current density of 0.1, 0.2, 0.3, 0.5, 1.0, and 2.0 A g^−1^. When the current density returns to 0.1 A g^−1^, the specific capacity is only 645.4 mAh g^−1^. Compared with the two materials with different structures, ZMO HMRs show better electrochemical properties than the ZMO-AC anode. More attractively, the rate capability achieved for ZMO HMRs is meaningfully better than those of other Mn-based metal oxides reported previously [38,39,40,43,44,45], especially at a high-rate density (Figure 5e).

To further explore the high current of the ZMO HMRs electrode material for rapid charge/discharge applications, the cycle stability of the ZMO HMRs anode under a high current is profiled in Figure 5f. The specific capacity of the ZMO HMRs can be maintained at 529 mAh g^−1^ after 1000 cycles at 2.0 A g^−1^. The superior electrochemical lithium storage performance of the porous ZMO HMRs can be primarily attributed to the unique microstructure. The synthesized porous hollow structure can not only greatly increase the contact area between the active material and the electrolyte, the active surface area of the electrode, and the lithium ion transport rate but also offer enough space to buffer the stress change during the lithiation/delithiation cycle and reduce the stress effect during the Li^+^ de-intercalation process, thus greatly increasing the cycle stability of the materials.

The EIS measurements of the ZMO HMRs and ZMO-AC electrodes were further performed to understand the excellent electrochemical behavior as profiled in Figure S10. In the typical Nyquist plots, the semicircle diameter represents the interface transfer resistance between the electrode material and the electrolyte, and the slope of the oblique line after the semicircle represents the impedance generated by the internal solid-phase diffusion of the active material, which is the Warburg impedance. As seen in Figure S10a and further fitted with the equivalent circuit in Figure S10b (the corresponding EIS-fitted data for the ZMO HMRs and ZMO-AC are listed in Table S2), the charge transfer impedance of the ZMO HMRs (62.3 Ω) is much smaller than ZMO-AC (86.6 Ω). Similarly, the slope of ZMO HMRS is significantly greater than that of ZMO-AC in the Warburg impedance region, suggesting a faster transport speed of ions in the electrode. Thus, the EIS analysis mentioned above can be well summarized as the porous hollow structure not only alleviates the volume expansion of the material but also shows full contact between the electrolyte and the active material, which shortens the ion/charge diffusion path and accelerates the transmission speed [38,39,43,46].

**Figure 6:**
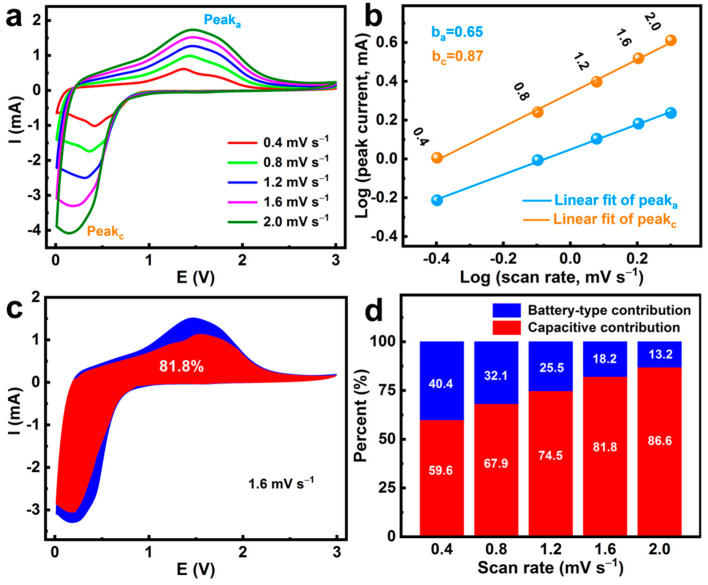
(**a**) CV curves with different scan rates, (**b**) log*i* vs. log*v* profiles, (**c**) the capacitive contribution (red region) and battery-type contribution (blue region) at 1.6 mV s^−1^, and (**d**) normalized capacitive (red region) contribution ratios at different scan rates.

To investigate the reasons for the excellent electrochemical lithium storage behavior of ZMO HMRs, kinetic analysis was performed with CV tests at various scan rates. Figure 6a displays the CV curve of the electrode at a scan rate of 0.4–2.0 mV s^−1^, where the relationship between the scan rate (*ν*) and the test current *(i*) can be expressed as [47,48,49]:

(5) $$i=av^{b}$$

(6) logi=blogv+loga

where *a* and *b* are the adjustment parameters; when *b* = 0.5, the charge storage is controlled by the diffusion of ions, and *b* = 1 primarily determines pseudocapacitive behavior. The calculated b value after fitting is shown in Figure 6b. The *b* value of *b_a_* = 0.65 corresponds to the oxidation peak, and *b_c_* = 0.87 corresponds to the reduction peak, indicating that the chemical lithium storage performance of ZMO HMRs is primarily attributed to the combined effect of battery behavior and pseudocapacitive behavior. In addition, the total capacitance contribution at different scan speeds can be divided into the pseudocapacitive capacity contribution *k_1_ν* and diffusion-controlled *k_2_ν^1/2^*, the relationship between which can be distinguished as the following equation [36,47,48,49]:

(7) $$i\left(V\right)=k_{1}v+k_{2}v^{1/2}$$

(8) $$i\left(V\right)/v^{1/2}=k_{1}v^{1/2}+k_{2}v$$

Specifically, *ν* is the scan rate at specific potentials, *V* is the specified potentials, and *k_1_* and *k_2_* are adjustable parameters, which can be obtained via linearly fitting *i(V)/ν^1/2^* and *ν^1/2^* under the specified potentials. The *k_1_ν* corresponds to the current contribution from the pseudocapacitive effects and *k_2_ν^1/2^* to the diffusion-controlled process, respectively. The capacitive contribution at a specific scan rate is calculated by dividing the area of the fitted closed curve by the area of the corresponding CV curve (Figure 6c). Similarly, the capacitive contribution at other scanning rates can also be calculated (Figure 6d).

Figure 6c provides a detailed quantitative capacitive contribution case of the ZMO HMRs electrode at 1.6 mV s^−1^. It can be clearly seen that the contribution of the capacitive capacity (the red region) accounts for approximately 88.4% of the whole capacity. The capacitance contributions at other scan rates are summarized in Figure 6d. It can be intuitively seen that the battery-type capacity contribution gradually decreases with the increase in the scan speed, and the corresponding capacitor capacity contribution gradually dominates. As the scan rate increases from 0.4, 0.8, 1.2, and 2.0 mV s^−1^, the capacitive contributions accordingly increase from 59.6% to 67.9%, 74.5%, and 86.6%, respectively. Such a result suggests that the excellent electrochemical performance of ZMO HMRs electrodes can be attributed to capacitive behavior rather than battery control. The hollow structure with rich pores provides efficient diffusion channels for Li^+^, reduces the diffusion path of Li^+^, and enables Li^+^ to react quickly, thus effectively improving the electrochemical performance accordingly.

**Figure 7:**
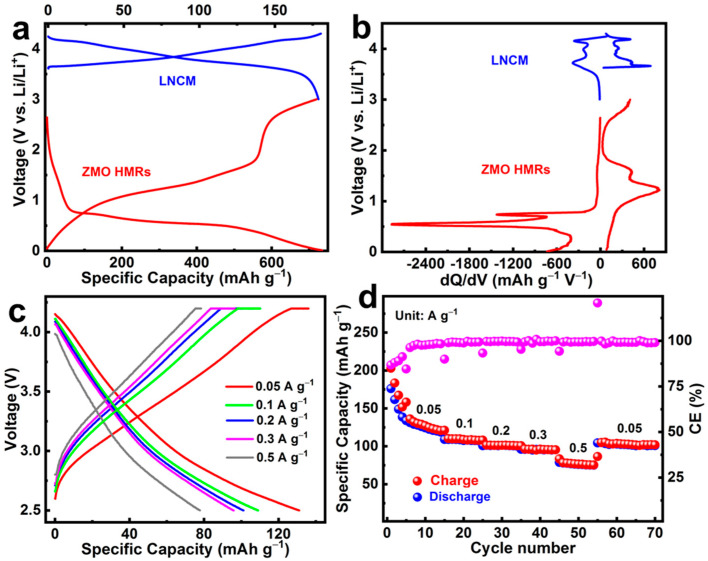
(**a**) The charge–discharge plots of LNCM and ZMO HMRs at 0.1 A g^−1^. (**b**) Corresponding *d*Q/*d*V curves of the LNCM and ZMO HMRs. (**c**) Charge and discharge profiles, and (**d**) rate capability of LNCM/ZMO HMRs full battery.

To further evaluate the electrochemical performance of ZMO HMRs for commercial application prospects, the full cell was assembled by using commercial LiNi_0.8_Co_0.1_Mn_0.1_O_2_ (LNCM) as a cathode and ZMO HMRs as the anode (Figure 7). The ZMO HMRs electrode was first pre-lithiated to eliminate the irreversible specific capacity of the first cycle, that is, the ZMO HMRs electrode and the metal lithium sheet were soaked directly in the electrolyte for 1 h. In the design process of the full battery, the anode electrode is excessive when avoiding the deposition of metallic lithium during the charging process and prevents the malfunction of batteries.

Figure 7a presents the initial charge/discharge curve of the LNCM/Li and ZMO HMRs/Li half-cell at the current density of 0.1 A g^−1^, and according to the specific capacity of the cathode and the anode, the capacity ratio ranged from 1: 1.1–1.5. Figure 7b shows the differential capacity–voltage diagrams corresponding to Figure 7a. Obviously, the average working potential to the redox process of Ni^2+^, Ni^3+^, and Ni^4+^ in LNCM is approximately 4.1 V, while the Zn^2+^ and Mn^2+^ in ZMO HMRs are approximately 0.7 V. Therefore, the average operating voltage of the combined device is designed as 3.4 V. Representative charge/discharge curves of the full cell are presented in Figure 7c. The discharge capacities were 131, 109, 101, 96, and 78 mAh g^−1^ at the current densities of 0.05, 0.1, 0.2, 0.3, and 0.5 A g^−1^, respectively. The specific capacity of the full cell decreases significantly in the first few cycles, which may be caused by the attenuation of the anode material in the initial cycle (Figure 7d). When the current density returns to 0.05 A g^−1^, the specific discharge capacity can still reach 105 mAh g^−1^ and remains at 101 mAh g^−1^ at the 70th cycle, indicating the excellent rate performance of the full cell. In addition, a material-level energy density of 273 Wh kg^−1^ was achieved corresponding to the current density of 0.05 A g^−1^. More importantly, the designed efficient strategy is also expected to be extended to the accurate synthesis of other polymetallic oxide materials, so as to provide ideal materials for electrochemical energy storage and other fields.

## 4. Conclusions

In summary, the porous ZMO HMRs were successfully fabricated by a facile co-precipitation reaction with chloride salt at room temperature and calcination at 400 °C for 2 h. Through exploring different experimental conditions, it is found that the content of polyethylene glycol 400 in the mixed solution plays a key role in the formation of the hollow structure. At the same time, different metal salts will lead to different microstructures, and only chloride salts lead to the formation of hollow precursors. More attractively, benefiting from the abundant multi-level pore structure and hollow structure, the ZMO HMRs anode exhibits excellent electrochemical lithium storage performance in terms of reversible capacity, rate capability, and long cycle life. In addition, the assembled ZMO HMRs-based full cells deliver an excellent rate capacity and high energy density. Such outstanding electrochemical performance enables the porous ZMO HMRs material to function as a promising anode material for high-power LIBs.

## Data Availability

The data presented in this study are available on request from the corresponding author.

## Supplementary Materials

The following supporting information can be downloaded at: https://www.mdpi.com/article/10.3390/nano13030512/s1, Figure S1: Rietveld-refined XRD pattern of ZnMn_2_O_4_. Table S1: Rietveld refinement results of XRD data for ZMO HMRs samples. Figure S2: (**a**,**b**) FESEM and (**c**,**d**) TEM images for the ZnC_2_O_4_-MnC_2_O_4_·2H_2_O precursor. Figure S3: TG-DSC curves of the ZnC_2_O_4_-MnC_2_O_4_·2H_2_O precursor. Figure S4: (**a**) Nitrogen adsorption/desorption isotherms and (**b**) pore-size distribution plot of the ZMO HMRs. Figure S5; The XRD pattern of ZMO-AC (**a**) and (**b**) ZMMO-NO. Figure S6: FESEM images of the ZMO-AC: (**a**) precursor, (**b**) after annealing, (**c**) TEM images for after annealing. ZMO-NO: (**d**) precursor, (**e**) after annealing, (**f**) TEM images for after annealing. Figure S7: Typical initial discharge-charge voltage profiles of the ZMO HMRs electrode and corresponding ex-situ XRD analysis at different selected voltages. Figure S8; FESEM image of the ZMO-HMRs cycled over 250 cycles. Figure S9: Cross-section SEM images of (**a**) original pristine ZMO HMRs electrode, (**b**) pristine ZMO HMRs electrode after 250 cycles at 0.5 A g^−1^. Figure S10: (**a**) EIS spectra and (**b**) corresponding equivalent circuit of ZMO HMRs and ZMO-AC. Table S2: Corresponding EIS fitted data for the ZMO HMRs and ZMO-AC.
