# Supplementary material for: Facile Construction of Porous ZnMn2O4 Hollow Micro-Rods as Advanced Anode Material for Lithium Ion Batteries
Source: Nanomaterials (Basel). 2023 Jan 27;13(3):512. doi: 10.3390/nano13030512 (PMC9920744; doi:10.3390/nano13030512)
Supplement: Supplementary file 1 [file nanomaterials-13-00512-s001.zip › nanomaterials-2171034-supplementary.pdf]

nanomaterials

*Supplementary Materials*

# Facile Construction of Porous $ZnMn_2O_4$ Hollow Micro-Rods as Advanced Anode Material for Lithium Ion Batteries

**Yuyan Wang †, Senyang Xu †, Yamin Zhang, Linrui Hou * and Changzhou Yuan ***

School of Materials Science & Engineering, University of Jinan, Jinan 250022, China
* Correspondence: mse_houlr@ujn.edu.cn (L.H.); mse_yuancz@ujn.edu.cn (C.Y.)
† Theses authors contributed equally to this work.

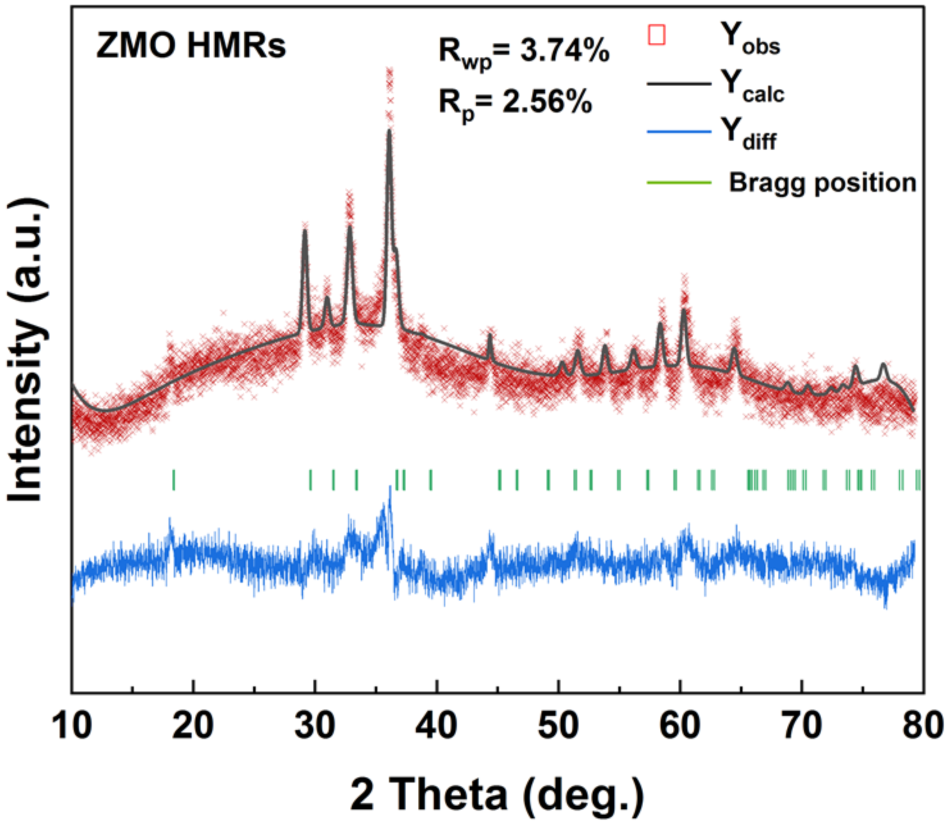


**Figure S1.** Rietveld-refined XRD pattern of $ZnMn_2O_4$.

**Table S1.** Rietveld refinement results of XRD data for ZMO HMRs samples.

| | a (Å) | b (Å) | c (Å) | volume (Å$^3$) | $R_{wp}$ (%) | $R_p$ (%) | CHI$^2$ |
|---|---|---|---|---|---|---|---|
| ZMO HMRs | 5.6533 | 5.6533 | 8.9989 | 287.612 | 3.74 | 2.56 | 2.882 |

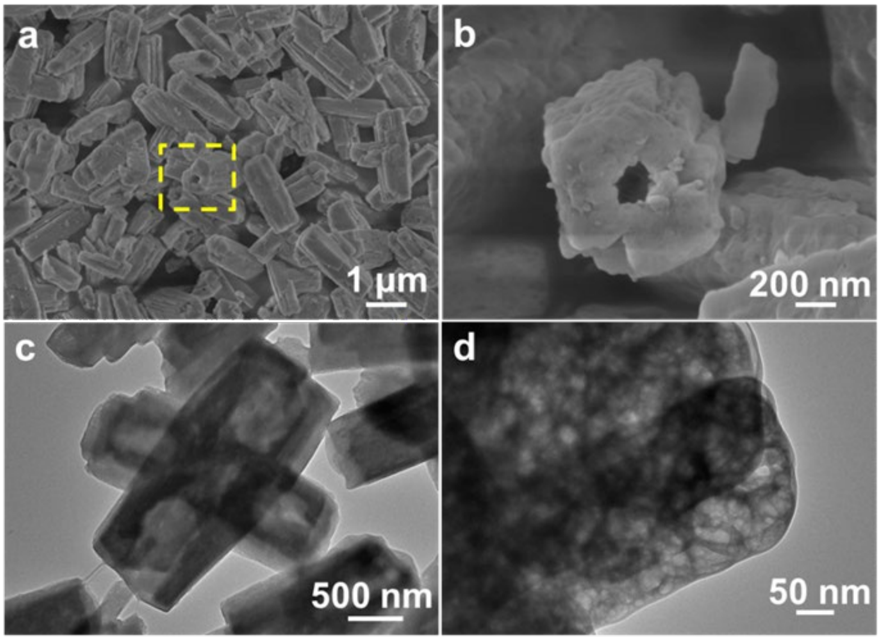


**Figure S2.** (**a**, **b**) FESEM and (**c**, **d**) TEM images for the $ZnC_2O_4$-$MnC_2O_4{\cdot}2H_2O$ precursor.

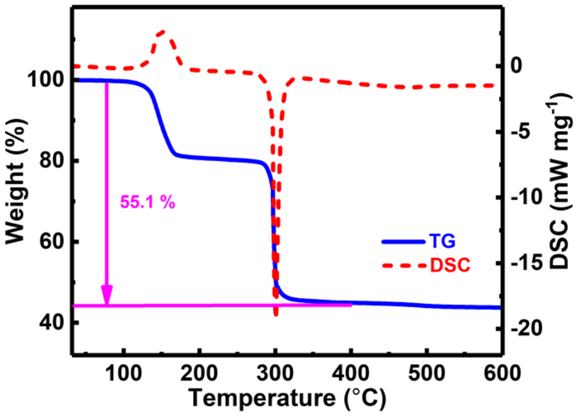


**Figure S3.** TG-DSC curves of the $ZnC_2O_4$-$MnC_2O_4{\cdot}2H_2O$ precursor.

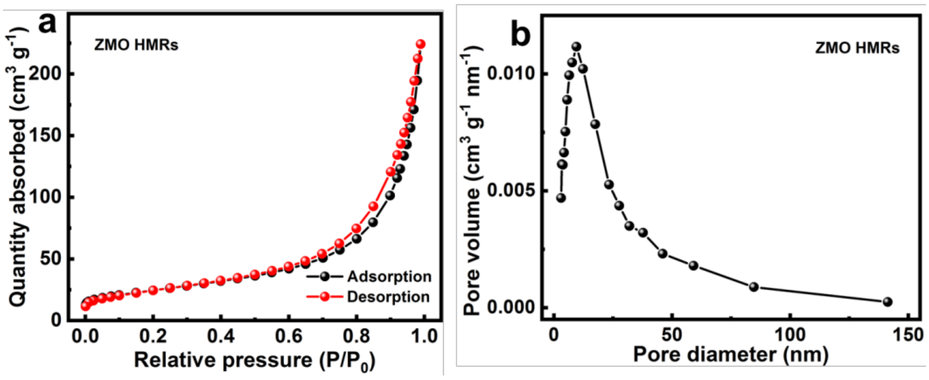


**Figure S4.** (**a**) Nitrogen adsorption/desorption isotherms and (**b**) pore-size distribution plot of the ZMO HMRs.

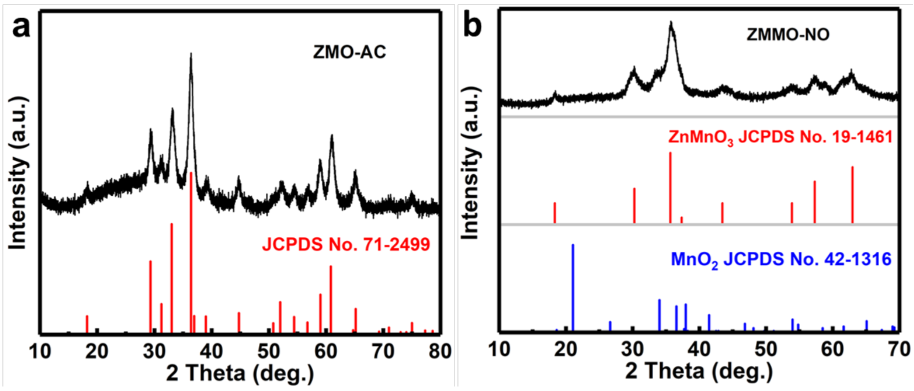


**Figure S5.** The XRD pattern of ZMO-AC (**a**) and (**b**) ZMMO-NO.

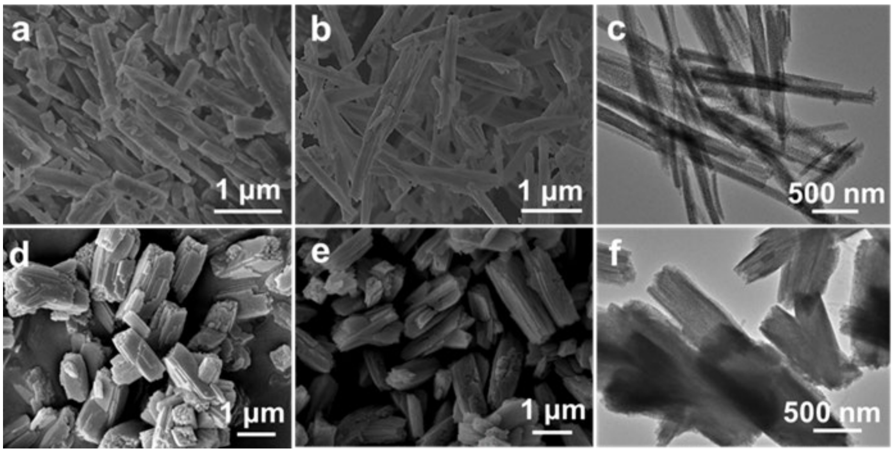


**Figure S6.** FESEM images of the ZMO-AC: (**a**) precursor, (**b**) after annealing, (**c**) TEM images for after annealing. ZMO-NO: (**d**) precursor, (**e**) after annealing, (**f**) TEM images for after annealing.

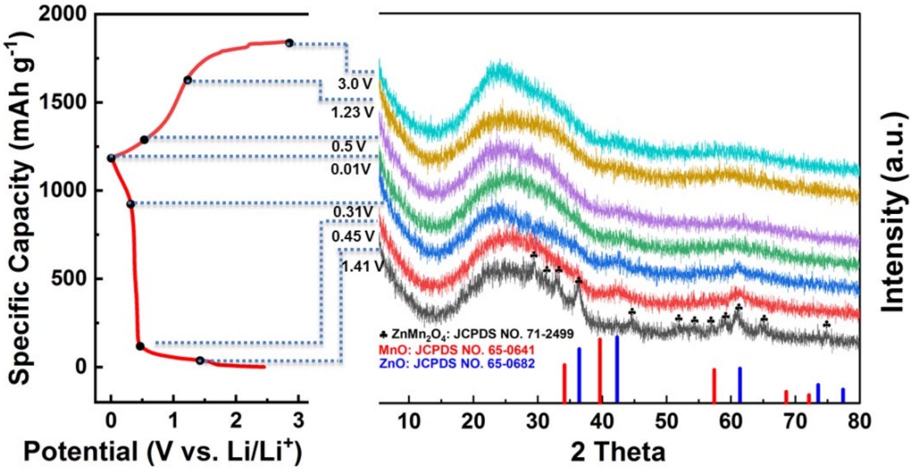


**Figure S7.** Typical initial discharge-charge voltage profiles of the ZMO HMRs electrode and corresponding ex-situ XRD analysis at different selected voltages.

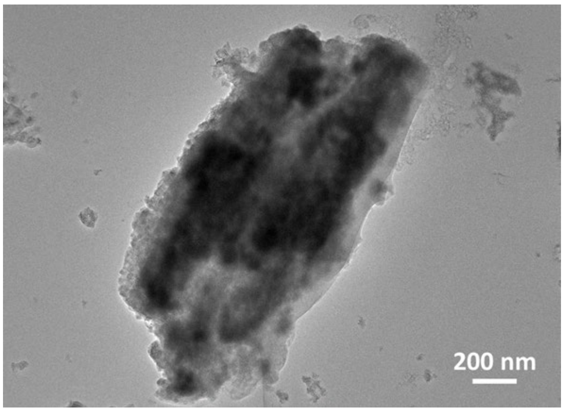


**Figure S8.** FESEM image of the ZMO-HMRs cycled over 250 cycles.

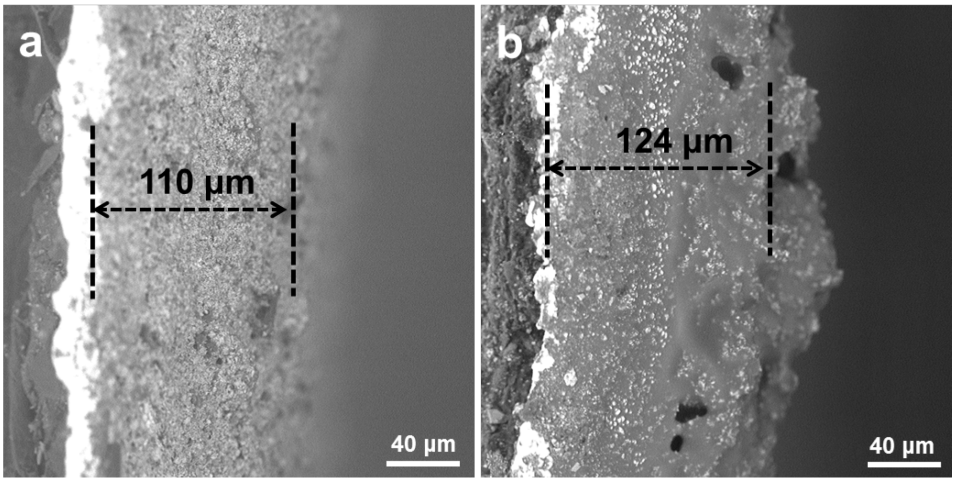


**Figure S9.** Cross-section SEM images of (**a**) original pristine ZMO HMRs electrode, (**b**) pristine ZMO HMRs electrode after 250 cycles at 0.5 A $g^{-1.}$

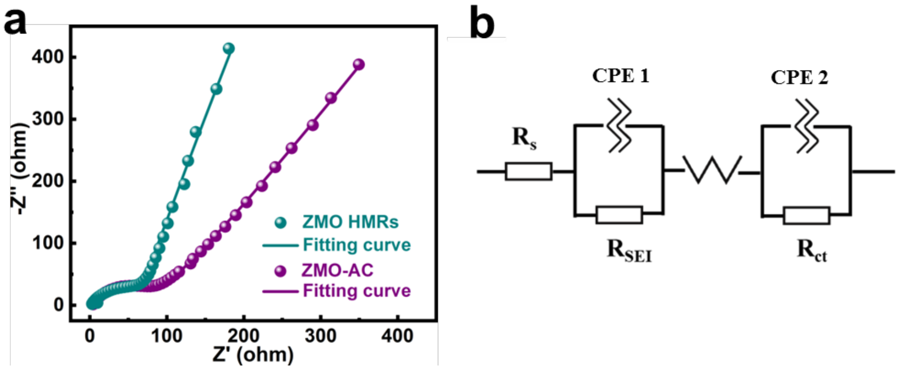


**Figure S10.** (**a**) EIS spectra and (**b**) corresponding equivalent circuit of ZMO HMRs and ZMO-AC.

**Table S2.** Corresponding EIS fitted data for the ZMO HMRs and ZMO-AC.

| Electrode | $R_S$ (Ω) | CPE 1 (Ω) | $R_{SEI}$ (Ω) | W (Ω) | CPE 2 (Ω) |
|---|---|---|---|---|---|
| ZMO HMRs | 1.616 | 0.6922 | 97.56 | 160.0 | 0.7896 |
| ZMO-AC | 3.183 | 0.6925 | 106.4 | 206.2 | 0.6719 |
